# Baseline and longitudinal changes in peak expiratory flow rate as predictors of sarcopenia in older adults: A 4-year cohort study

**DOI:** 10.1016/j.jnha.2025.100640

**Published:** 2025-07-24

**Authors:** Hui Qian, Qifeng Chen, Kangkang Chen

**Affiliations:** a Rehabilitation Medicine Department, The Affiliated Hospital of Shaoxing University, Shaoxing, China; b Department of Non-Communicable Diseases Control and Prevention, Shaoxing Center for Disease Control and Prevention, Shaoxing, China

**Keywords:** CHARLS, Older adults, Peak expiratory flow rate, Sarcopenia

## Abstract

**Objective:**

Peak expiratory flow rate (PEFR) is a cheap and simple tool for assessing airway patency and respiratory muscle strength. So far, the impact of PEFR, particularly its longitudinal changes, on the prevalence and incidence of sarcopenia remains underexplored. Therefore, we conducted a cross-sectional and longitudinal study to address this gap.

**Methods:**

We analyzed data from the China Health and Retirement Longitudinal Study (CHARLS). A total of 5,280 older adults were selected as a cohort in 2011, of whom 3,686 were confirmed sarcopenia-free at baseline and followed through 2015. Longitudinal changes in PEFR were measured in 2011 and 2013, with complete paired data available for 2,904 subjects. Sarcopenia was diagnosed according to the 2019 Asian Working Group for Sarcopenia (AWGS). Multivariable logistic regression and discrete-time proportional hazards models were used to assess associations between baseline PEFR, 2-year PEFR changes, and sarcopenia risk, adjusting for potential confounders.

**Results:**

A 1-standard deviation (SD) decrease in baseline PEFR was associated with 56% higher odds of prevalent sarcopenia (OR = 1.56, 95% CI = 1.38−1.75), and PEFR (% predicted) <80% with 93% higher odds (OR = 1.93, 95% CI = 1.49–2.50). Over the 4-year follow-up, these reductions were linked to increased risk of incident sarcopenia (HR = 1.26, 95% CI = 1.13−1.40, and HR = 1.47, 95% CI = 1.17−1.84, respectively). A decline from PEFR (% predicted) ≥80% to <80% was associated with 120% higher odds (OR = 2.20, 95% CI = 1.31−3.71), while improvement from <80% to ≥80% was linked to 30% lower odds (OR = 0.70, 95% CI = 0.50−0.96).

**Conclusions:**

Lower baseline PEFR and its longitudinal decline were associated with increased risk of sarcopenia, while upward changes were linked to lower risk. These findings suggest that PEFR may serve as a practical early marker for identifying older adults at elevated risk of sarcopenia.

## 1 Introduction

Sarcopenia is an age-related geriatric syndrome characterized by progressive decline in muscle mass, strength, and/or physical performance [1,2]. A recent meta-analysis has found that its prevalence ranges from 10% to 27% in individuals aged 60 years and older globally [3]. Older adults with sarcopenia often face multiple adverse clinical events, including falls, loss of functional capacity, frailty, and increased mortality [[4], [5], [6], [7]], imposing substantial burdens on both individuals and healthcare systems, especially as the global population continues to age [8]. Fortunately, sarcopenia is potentially reversible with early and targeted interventions [9,10], highlighting the clinical value of identifying reliable early indicators. Peak expiratory flow rate (PEFR), a simple and non-invasive pulmonary function parameter, has emerged as a potential candidate biomarker.

PEFR, defined as the maximal airflow during forced exhalation, also declines with age and reflects both pulmonary function and respiratory muscle strength [11,12]. Several studies have suggested that lower PEFR may precede musculoskeletal decline, being associated with slower gait speed and loss of muscle mass [13,14]. Furthermore, both cross-sectional studies indicate an inverse association between PEFR and sarcopenia prevalence [15,16], while a 4-year longitudinal study by Hu et al. [17] found that each one standard deviation (SD) increase in baseline PEFR was associated with a 28% lower likelihood of incident sarcopenia. However, these studies focused solely on static baseline PEFR values without considering longitudinal changes. Observing changes in PEFR over time may provide additional insights into early physiological alterations associated with sarcopenia risk. Such dynamic trends might reflect underlying pathophysiological processes that precede clinical manifestations. Additionally, previous studies were limited by single-design approaches: cross-sectional analyses lack temporal directionality, while longitudinal studies may fail to capture concurrent physiological associations.

To address these gaps, we conducted an integrated analysis using both cross-sectional and longitudinal data from the same cohort to examine: (1) the association between baseline PEFR and sarcopenia prevalence in 2011; (2) the relationship between baseline PEFR and 4-year incident sarcopenia; and (3) particularly the association between 2-year PEFR changes and subsequent risk of sarcopenia. This work may help evaluate the utility of PEFR as a clinically accessible early predictor of sarcopenia, supporting improved risk stratification and prevention strategies for older adults.

## 2 Methods

### 2.1 Study participants

Data for this study was sourced from the China Health and Retirement Longitudinal Study (CHARLS), a cohort established in 2011. The study surveyed Chinese adults aged 45 and older from 450 villages, 150 counties, and 28 provinces, representing diverse social and geographic backgrounds. The initial sample included 17,708 adults, who were followed up every 2–3 years. The study collects a wide range of variables, including demographic details, lifestyles, health conditions, physical measurements, and so on. Detailed descriptions of the CHARLS can be found in earlier studies [18]. The protocol was approved by the Ethical Review Committee of Peking University (IRB 00001052-11015). All participants provided written informed consent before the examination.

**Fig. 1:**
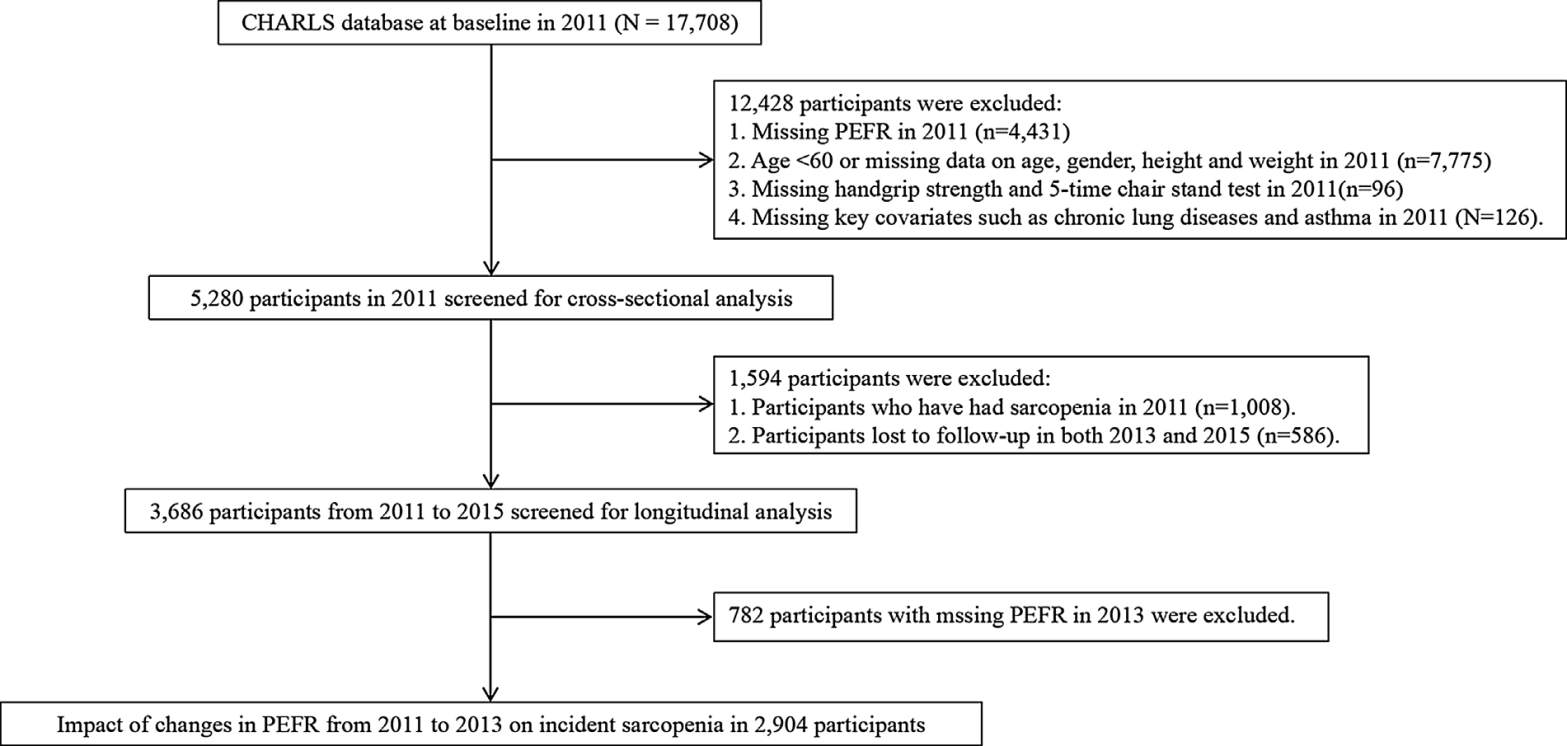
Flowchart of the sample selection process.

So far, the CHARLS project has released five waves of data (2011, 2013, 2015, 2018, 2020). However, as physiological indicators (e.g., PEFR and handgrip strength) and anthropometric measurements (e.g., height and weight) were only collected in 2011, 2013, and 2015, our analysis was confined to these three waves. Exclusion criteria included missing age data, participants younger than 60, and missing key variables (e.g., gender, weight, height, PEFR, handgrip strength, 5-time chair stand test, and other covariates) in 2011. This resulted in 5,280 older adults eligible for the cross-sectional analysis. For the longitudinal analysis, we excluded those with pre-existing sarcopenia in 2011 and the participants lost to follow-up in both 2013 and 2015, leaving 3,686 individuals eligible for the 4-year longitudinal analysis. Finally, for the analysis of PEFR changes and incident sarcopenia, we excluded participants with missing PEFR data in 2013, resulting in 2,904 eligible participants. The detailed screening process is presented in Fig. 1.

### 2.2 Peak expiratory flow rate measurement

Older adults were instructed to stand upright, take a deep breath, and then exhale forcefully and quickly into a peak flow meter (Everpure™ Peak Flow Meter, with a disposable mouthpiece, Shanghai, China). The PEFR was measured three times, and the highest value was selected. Predicted PEFR values were calculated using separate formulas for men and women: for men, PEFR (L/s) = 21.566 + 0.078 × H − 6.192 × ln(A), and for women, PEFR (L/s) = −22.741 + 6.901 × ln(H) − 0.090 × A, where A is age in years and H is height in centimeters [19]. These formulas were developed on the basis of Chinese older adults aged 60–84 from Jinan, which closely matches the characteristics of our study population, thereby reducing potential bias from demographic differences. PEFR (% predicted) was calculated as the percentage of the actual value relative to the predicted PEFR. A value less than 80% was considered indicative of impaired airway patency and low respiratory muscle strength [20,21].

### 2.3 Muscle strength measurement

Grip strength was used to indicate muscle strength. Older adults were instructed to stand, grasp a mechanical dynamometer (Yuejian™ WL-1000 dynamometer, Nantong, China) with both hands at a right angle and squeeze as forcefully as possible for several seconds. Each hand was tested twice, and the highest of the four measurements was recorded. Low muscle strength was defined as a grip strength of <28 kg for men and <18 kg for women [2].

### 2.4 Physical performance measurement

Five-time chair stand test was used to indicate physical performance. Older adults were instructed to sit (chair height was adjusted according to participants' stature) with their arms crossed over their chest, then stand up and sit down as quickly as possible, repeating the action five times without pausing or using their arms for support. The investigators recorded the total time taken to complete the task. Older adults who took 12 seconds or more to finish the test were classified as having low physical performance [2].

### 2.5 Muscle mass measurement

Since CHARLS did not directly measure muscle mass, we estimated it using a formula validated by multiple studies [[22], [23], [24]], which demonstrated strong correlation with dual-energy X-ray absorptiometry (DXA) measurements. The formula for calculating appendicular skeletal muscle (ASM) mass was as follows: ASM = 0.193 × weight (kg) + 0.107 × height (cm) − 4.157 × sex − 0.037 × age (years) − 2.631, where sex is coded as 1 for men and 2 for women. ASM was then adjusted for height (ASM/Ht^2^). According to the 2019 Asian Working Group for Sarcopenia (AWGS), low ASM mass is defined as <7.0 kg/m² for men and <5.4 kg/m² for women [2].

### 2.6 Outcome definition

According to the AWGS 2019 [2], sarcopenia was defined as the presence of low ASM mass combined with either low muscle strength or low physical performance.

### 2.7 Covariates

To minimize the impact of confounding factors, a set of potential covariates was selected, including age, sex, type of residence, marital status, education, smoking, alcohol consumption, body mass index (BMI), complete tooth loss, a 6-item activities of daily living (ADL) scale (assessing difficulties in bathing, dressing, eating, getting in/out of bed, toileting, and urinary continence), participation in physical activities like Tai Chi or park-based exercise (defined as participation at least once in the past month), comorbidities (including chronic lung diseases, asthma, diabetes, heart problems, stroke, cancer, kidney disease, liver disease, arthritis, hypertension, and digestive diseases), and the number of medications taken.

### 2.8 Statistical analysis

Baseline characteristics of participants were summarized as means ± SD for continuous variables and numbers (percentages) for categorical variables, stratified by PEFR (% predicted) groups. Between-group differences were assessed using one-way analysis of variance (ANOVA) for continuous variables and chi-squared tests for categorical variables. To examine the association between baseline PEFR (per 1 SD decrease, standardized based on the distribution in the overall cohort), baseline PEFR (% predicted) and the prevalence of sarcopenia, odds ratios (ORs) and their 95% confidence intervals (95% CI) were calculated using multivariable logistic regression models, adjusting for potential confounders. The proportional hazards assumption was checked using the Kaplan–Meier estimator, and survival functions were compared with the log-rank test. Since the surveys were conducted every two years, multivariable discrete-time complementary log–log (CLL) proportional hazards models were employed to investigate the associations between baseline PEFR (per 1 SD decrease, standardized based on the distribution in the overall cohort), baseline PEFR (% predicted), and the development of sarcopenia from 2011 to 2015. Similar methods were used to analyze the association with changes in PEFR (% predicted). In addition to the main analyses, subgroup analyses were performed separately for two PEFR variable types. For PEFR (per 1 SD decrease), participants were stratified into four sex- and age-defined subgroups (<75 vs. ≥75 years), with PEFR standardized within each subgroup (z-scores); associations with both prevalent and incident sarcopenia were then examined using multivariable models. For PEFR (% predicted), subgroup analyses assessed potential interactions with demographic and clinical characteristics in relation to sarcopenia risk. All data analyses were performed using R version 4.3.0, with a significance level set at *P* < 0.05 for two-tailed tests.

## 3 Results

### 3.1 Baseline characteristics of the study population

A total of 5,280 eligible older adults were included in the baseline analysis (Table 1). Those with PEFR (% predicted) ≥80% had higher BMI, were more likely to live in urban areas, have higher education, and show lower prevalence of smoking, chronic lung disease, and asthma. They also used fewer medications, experienced fewer ADL difficulties, had lower rates of complete tooth loss, and engaged more in physical activities. In contrast, older adults identified as PEFR (%predicted) <80% had higher levels of each of the sarcopenia components (low ASM mass, low handgrip strength, low physical performance; all *P* < 0.001).

**Table 1:** Baseline characteristics of older adults by peak expiratory flow rate (% predicted) classification in 2011.

Variables	Overall (n = 5,280)	PEFR (%predicted) ≥80% (n = 1,157)	PEFR (%predicted) <80% (n = 4,123)	Statistic	*P*
Age, mean ± SD (years)	67.72 ± 6.39	67.71 ± 6.54	67.73 ± 6.34	t=-0.08	0.934
Sex (%)				χ² = 1.72	0.189
Men	2,714 (51.40)	575 (49.70)	2,139 (51.88)		
Women	2,566 (48.60)	582 (50.30)	1,984 (48.12)		
Height, mean ± SD (m)	156.65 ± 8.78	157.05 ± 8.69	156.54 ± 8.80	t = 1.76	0.079
BMI, mean ± SD (kg/m^2^)	22.93 ± 3.72	23.56 ± 3.66	22.76 ± 3.71	t = 6.54	**<.001**
Type of residence (%)				χ² = 13.37	**<.001**
Urban Community	1,944 (36.82)	479 (41.40)	1,465 (35.53)		
Rural Village	3,336 (63.18)	678 (58.60)	2,658 (64.47)		
Marital status (%)				χ² = 1.22	0.270
Married/married but separated	4,206 (79.66)	935 (80.81)	3,271 (79.34)		
Unmarried/divorced/widowed	1,074 (20.34)	222 (19.19)	852 (20.66)		
Education levels (%)				χ² = 82.19	**<.001**
No formal education	2,971 (56.27)	541 (46.76)	2,430 (58.94)		
Primary school	1,369 (25.93)	324 (28.00)	1,045 (25.35)		
Middle school	621 (11.76)	174 (15.04)	447 (10.84)		
High school or above	319 (6.04)	118 (10.20)	201 (4.88)		
Smoking status (%)				χ² = 10.08	**0.002**
No	2,997 (56.76)	704 (60.85)	2,293 (55.61)		
Yes	2,283 (43.24)	453 (39.15)	1,830 (44.39)		
Alcohol consumption (%)				χ² = 2.74	0.098
No	3,124 (59.17)	709 (61.28)	2,415 (58.57)		
Yes	2,156 (40.83)	448 (38.72)	1,708 (41.43)		
Chronic lung disease (%)				χ² = 78.33	**<.001**
No	4,575 (86.65)	1,093 (94.47)	3,482 (84.45)		
Yes	705 (13.35)	64 (5.53)	641 (15.55)		
Asthma (%)				χ² = 61.34	**<.001**
No	4,938 (93.52)	1,140 (98.53)	3,798 (92.12)		
Yes	342 (6.48)	17 (1.47)	325 (7.88)		
Diabetes (%)				χ² = 1.19	0.276
No	4,877 (92.37)	1,060 (91.62)	3,817 (92.58)		
Yes	403 (7.63)	97 (8.38)	306 (7.42)		
Heart Problem (%)				χ² = 1.92	0.166
No	4,444 (84.17)	989 (85.48)	3,455 (83.80)		
Yes	836 (15.83)	168 (14.52)	668 (16.20)		
Stroke (%)				χ² = 2.38	0.123
No	5,090 (96.40)	1,124 (97.15)	3,966 (96.19)		
Yes	190 (3.60)	33 (2.85)	157 (3.81)		
Cancer (%)				χ²=0.34	0.559
No	5,237 (99.19)	1,146 (99.05)	4,091 (99.22)		
Yes	43 (0.81)	11 (0.95)	32 (0.78)		
Kidney disease (%)				χ² = 3.25	0.072
No	4,971 (94.15)	1,102 (95.25)	3,869 (93.84)		
Yes	309 (5.85)	55 (4.75)	254 (6.16)		
Liver disease (%)				χ² = 1.23	0.268
No	5,102 (96.63)	1,124 (97.15)	3,978 (96.48)		
Yes	178 (3.37)	33 (2.85)	145 (3.52)		
Arthritis (%)				χ²=0.26	0.611
No	3,324 (62.95)	721 (62.32)	2,603 (63.13)		
Yes	1,956 (37.05)	436 (37.68)	1,520 (36.87)		
Hypertension (%)				χ²=0.64	0.425
No	3,538 (67.01)	764 (66.03)	2,774 (67.28)		
Yes	1,742 (32.99)	393 (33.97)	1,349 (32.72)		
Digestive disease (%)				χ² = 2.37	0.124
No	4,139 (78.39)	926 (80.03)	3,213 (77.93)		
Yes	1,141 (21.61)	231 (19.97)	910 (22.07)		
Number of medications (%)				χ² = 17.17	**<.001**
0	2,409 (45.62)	563 (48.66)	1,846 (44.77)		
1	1,592 (30.15)	367 (31.72)	1,225 (29.71)		
≥2	1,279 (24.22)	227 (19.62)	1,052 (25.52)		
Activities of daily living (%)				χ² = 33.28	**<.001**
0	4,082 (77.31)	966 (83.49)	3,116 (75.58)		
1	626 (11.86)	107 (9.25)	519 (12.59)		
≥2	572 (10.83)	84 (7.26)	488 (11.84)		
Complete tooth loss (%)				χ² = 12.76	**<.001**
No	4,419 (83.69)	1,008 (87.12)	3,411 (82.73)		
Yes	861 (16.31)	149 (12.88)	712 (17.27)		
Physical activities (%)				χ² = 14.41	**<.001**
No	2,510 (47.54)	607 (52.46)	1,903 (46.16)		
Yes	2,770 (52.46)	550 (47.54)	2,220 (53.84)		
Low ASM mass (%)				χ² = 32.17	**<.001**
No	2,510 (47.54)	844 (72.95)	2,639 (64.01)		
Yes	2,770 (52.46)	313 (27.05)	1,484 (35.99)		
Low handgrip strength (%)				χ² = 66.87	**<.001**
No	4,260 (81.91)	1,037 (90.10)	3,223 (79.58)		
Yes	941 (18.09)	114 (9.90)	827 (20.42)		
Low physical performance (%)				χ² = 60.90	**<.001**
No	2,992 (60.53)	787 (70.58)	2,205 (57.60)		
Yes	1,951 (39.47)	328 (29.42)	1,623 (42.40)		
Sarcopenia (%)				χ² = 50.42	**<.001**
No	4,272 (80.91)	1,020 (88.16)	3,252 (78.87)		
Yes	1,008 (19.09)	137 (11.84)	871 (21.13)		
Raw PEFR, mean ± SD (l/min)	254.55 ± 116.82	394.76 ± 92.80	215.21 ± 89.42	t = 59.85	**<.001**

PEFR, peak expiratory flow rate; SD, standard deviation; BMI, body mass index; ASM, appendicular skeletal muscle mass.

### 3.2 Associations of baseline PEFR with prevalent and incident sarcopenia

In 2011, a total of 1,008 older adults (19.1%) had prevalent sarcopenia. According to the multivariable logistic regression model, a one SD reduction in PEFR was linked to a 1.56-fold increase in the odds of prevalent sarcopenia (OR = 1.56, 95% CI = 1.38–1.75, *P* < 0.001) (Table 2). Sensitivity analyses stratified by sex and age (with within-group PEFR standardization) yielded consistent results (Table S1). Compared to older adults in the PEFR (% predicted) ≥80% reference group, those with PEFR (% predicted) <80% showed 1.93 times higher odds of prevalent sarcopenia (OR = 1.93, 95% CI = 1.49–2.50, *P* < 0.001). No significant interactions were found between PEFR (% predicted) classification and potential confounders (all *P* for interaction > 0.05; Table S2).

**Table 2:** Association between peak expiratory flow rate and sarcopenia.

	Prevalent sarcopenia＃				Incident sarcopenia§			
	Model 1		Model 2		Model 1		Model 2	
	OR (95% CI)	*p*-value	OR (95% CI)	*p*-value	HR (95% CI)	*p*-value	HR (95% CI)	*p*-value
PEFR continuous (1 SD decrease)	1.60 (1.46−1.76)	**<0.001**	1.56 (1.38−1.75)	**<0.001**	1.32 (1.19−1.46)	**<0.001**	1.26 (1.13−1.40)	**<0.001**
PEFR (% predicted)								
<80	2.01 (1.62−2.49)	**<0.001**	1.93 (1.49−2.50)	**<0.001**	1.56 (1.25−1.97)	**<0.001**	1.47 (1.17−1.84)	**<0.001**
≥80	Reference		Reference		Reference		Reference	

PEFR, peak expiratory flow rate; OR, odds ratio; HR, hazard ratio; CI, confidence interval; SD, standard deviation.

Model 1 adjusted for age, sex, marital status, education levels, type of residence, smoking, alcohol consumption, physical activities, complete tooth loss, and activities of daily living;

Model 2 adjusted for model 1 plus body mass index, chronic lung disease, asthma, diabetes, heart problem, stroke, cancer, kidney disease, liver disease, arthritis, hypertension, digestive disease, and number of medications.

＃ Logistic regression models of cross-sectional CHARLS 2011 data (n = 5,280).

§ Discrete-time complementary log-log proportional hazards models of longitudinal CHARLS 2011–2015 data (n = 3,686).

After excluding participants with sarcopenia at baseline and those lost to follow-up, 3,686 older adults remained. Over the 4-year follow-up period, 525 (14.2%) developed sarcopenia. Kaplan–Meier analysis indicated that 4-year incidence of sarcopenia was significantly higher in older adults with PEFR (% predicted) <80% (HR = 1.53, 95% CI = 1.22–1.92, *P* < 0.001) (Fig. S1). After adjusting for confounding factors, multivariable discrete-time CLL proportional hazards models demonstrated that a one SD reduction in PEFR was associated with a 1.26-fold increase in the hazard of incident sarcopenia (HR = 1.26, 95% CI = 1.13–1.40, *P <* 0.001) (Table 2). Sensitivity analyses stratified by sex and age (with within-group PEFR standardization) showed consistent associations in the <75-year subgroups, whereas associations in the ≥75-year groups were not statistically significant, likely due to smaller sample sizes (Table S3). Compared to older adults with PEFR (% predicted) ≥80%, those with PEFR (% predicted) <80% showed 1.47 times higher hazards of incident sarcopenia (HR = 1.47, 95% CI = 1.17–1.84, *P* < 0.001). The strength of association between low PEFR and incident sarcopenia was greater among older adults living in urban areas (*P* for interaction = 0.005) and those not using medication (*P* for interaction = 0.010) (Table S4).

### 3.3 Association of changes in PEFR with incident sarcopenia

**Fig. 2:**
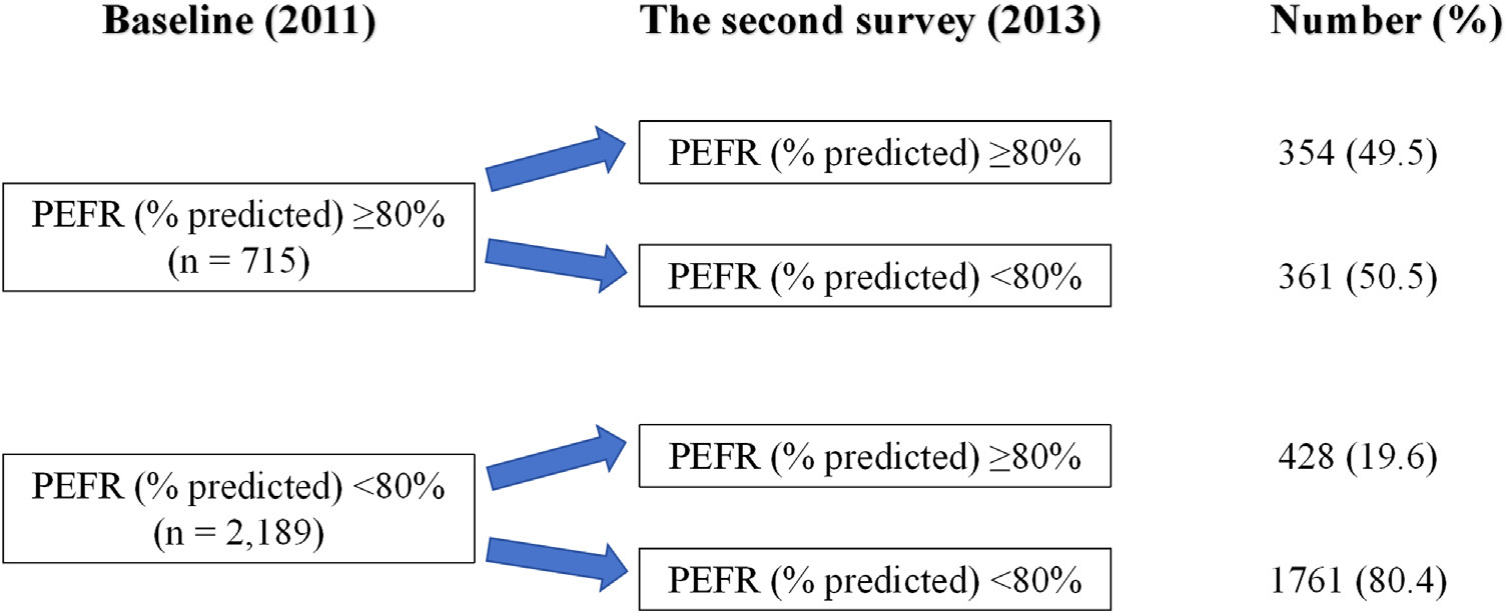
Number and percentage of the changes in PEFR (% predicted) classification (PEFR: peak expiratory flow rate).

Fig. 2 shows the number and percentage of older adults whose PEFR (% predicted) classification changed during the 2-year follow-up period. Among older adults with a baseline PEFR (% predicted) ≥80%, 361 participants (50.5%) experienced a decline to PEFR (% predicted) <80%. In contrast, among those with a baseline PEFR (% predicted) <80%, 428 (19.6%) improved to PEFR (% predicted) ≥80%. Table 3 presents the associations between changes in PEFR classification and incident sarcopenia risk. Compared to older adults with stable PEFR (% predicted) ≥80%, those who declined to PEFR (% predicted) <80% had a significantly higher risk of incident sarcopenia (HR = 2.20, 95% CI = 1.31–3.71, *P =* 0.003). In contrast, older adults with a baseline PEFR (% predicted) <80% who transitioned to PEFR (% predicted) ≥80% were less likely to develop sarcopenia compared to those with stable PEFR (% predicted) <80% (HR = 0.70, 95% CI = 0.50−0.96, *P =* 0.028). No significant interactions were found between changes in PEFR (% predicted) classification and potential confounders regarding incident sarcopenia (all *P* for interaction > 0.05; Table S5).

**Table 3:** Association between changes in peak expiratory flow rate status and incident sarcopenia (n = 2,904).

Status		Crude model		Model 1		Model 2		Model 3	
	sarcopenia/n	OR (95% CI)	*P*	OR (95% CI)	*P*	OR (95% CI)	*P*	OR (95% CI)	*P*
Stable PEFR (% predicted) ≥80%	24/354	Reference		Reference		Reference		Reference	
PEFR (% predicted) ≥80% to PEFR (% predicted) <80%	58/361	2.39 (1.49−3.85)	**<.001**	2.40 (1.47−3.93)	**<.001**	2.32 (1.39−3.88)	**0.001**	2.20 (1.31−3.71)	**0.003**
Stable PEFR (% predicted) <80%	299/1,761	Reference		Reference		Reference		Reference	
PEFR (% predicted) <80% to PEFR (% predicted) ≥80%	44/428	0.58 (0.42−0.80)	**<.001**	0.62 (0.45−0.86)	**0.004**	0.68 (0.49−0.93)	**0.017**	0.70 (0.50−0.96)	**0.028**

PEFR, peak expiratory flow rate; HR, hazards ratio; CI, confidence interval.

Model 1 adjusted for age, sex, marital status, education levels, and type of residence;

Model 2 adjusted for model 1 plus smoking, alcohol consumption, body mass index, physical activities, complete tooth loss, and activities of daily living;

Model 3 adjusted for model 2 plus chronic lung disease, asthma, diabetes, heart problem, stroke, cancer, kidney disease, liver disease, arthritis, hypertension, digestive disease, and number of medications.

## 4 Discussion

This study examined the relationship between baseline PEFR, changes in PEFR, and the risk of sarcopenia using data from a large cohort of community-dwelling older adults. Logistic regression showed that a one SD decrease in baseline PEFR and a baseline PEFR (% predicted) <80% were associated with a 56% and 93% increase in the odds of prevalent sarcopenia, respectively. After 4 years of follow-up, these reductions were also linked to 26% and 47% higher hazards of incident sarcopenia. Importantly, participants whose PEFR (% predicted) declined from ≥80% to <80% had a 120% higher risk of sarcopenia, while those who transitioned from <80% to ≥80% had a 30% lower risk. These findings highlight PEFR as a potential dynamic marker for identifying older adults at elevated risk of sarcopenia.

Given that PEFR serves as a clinical indicator of airway patency, the observed association may originate from impaired airway function. This impairment may be physiological, resulting from decreased airway elasticity with age, or pathological, as seen in respiratory diseases such as asthma and chronic obstructive pulmonary disease. Whether physiological or pathological, both forms of impaired airway patency can reduce tissue oxygenation efficiency and induce chronic hypoxia. Hypoxic conditions have been shown to impair muscle metabolism, promote myotube atrophy, and accelerate muscle degradation processes [25,26]. Additionally, restricted airflow can limit physical capacity, increasing sedentary behavior—a known contributor to muscle loss [27,28]. Pulmonary impairment is also often associated with elevated pro-inflammatory cytokines, such as IL-6 and TNF-α, which have been implicated in the pathogenesis of sarcopenia [29,30].

Respiratory muscle weakness may represent an additional pathway linking low PEFR with sarcopenia. During forced exhalation, the diaphragm and other accessory muscles are actively engaged. A low PEFR in the absence of airway obstruction may indicate weakened respiratory muscles. In this context, PEFR has been used as a surrogate for respiratory muscle strength [12,31]. In older adults, respiratory muscle weakness can impair the clearance of foreign pathogens, inflammation, secretions, and allergens, potentially increasing susceptibility to respiratory diseases, thereby triggering further muscle wasting. For instance, Okazaki et al. [32] found that respiratory muscles weakness was significantly associated with a higher risk of pneumonia. In turn, respiratory diseases can exacerbate muscle decline through systemic inflammation [29,30], shortness of breath [33], and mechanical ventilation-associated atrophy [34,35]. On the other hand, due to shared pathophysiological mechanisms such as mitochondrial dysfunction [36], the age-related decline of respiratory muscles—integral components of the skeletal muscle system—may signal systemic muscle deterioration. Previous studies have shown that reduced respiratory muscle strength correlates with decreased limb muscle strength and skeletal muscle mass [37]. In our study, after adjusting for chronic respiratory diseases in multivariable models, the association between low PEFR and increased sarcopenia risk remained significant, further supporting this mechanism. Additionally, Hu et al. [17] found that cognition mediates the relationship between low PEFR and sarcopenia risk, although cognition explains only one-tenth of this association. This suggests that there are also other mechanisms contributing to the link between low PEFR and sarcopenia, warranting further investigation.

As sarcopenia emerges as a significant public health concern, the findings of this study carry important clinical implications. First, current research on the association between PEFR and sarcopenia remains limited. We confirm that low baseline PEFR is associated with an elevated risk of both incident and prevalent sarcopenia, in line with previous observational studies [[15], [16], [17]], thereby strengthening the current evidence base. As a cheap, simple, and non-invasive tool, PEFR monitoring can be incorporated into routine geriatric assessments to facilitate early identification of older adults at increased risk. Those with reduced PEFR values could be considered for prioritized preventive monitoring. Notably, stratified analyses revealed stronger associations between low PEFR and incident sarcopenia among urban residents and those not taking medications. Environmental exposures such as air pollution, combined with reduced access to exercise facilities, may exacerbate vulnerability in urban settings [38,39]. In contrast, rural older adults may stay physically active through daily labor, which could help preserve muscle mass even in the context of reduced PEFR. Similarly, individuals not on medications may lack adequate disease management, allowing symptoms like dyspnea or fatigue to persist, thereby reducing physical activity and accelerating muscle wasting. These subgroup-specific findings indirectly support the hypothesis that PEFR and sarcopenia may share common etiological pathways, underscoring the potential of PEFR as an early indicator and the need for targeted prevention in vulnerable populations.

Second, beyond baseline PEFR, we examined whether longitudinal changes in PEFR are indicative of future risk of sarcopenia, a topic not previously explored. Our findings suggest that declines in PEFR over time are associated with increased risk of subsequent sarcopenia, while improvements in PEFR are associated with a lower risk. Notably, participants whose PEFR (% predicted) dropped from ≥80% to <80% had a 120% higher risk of sarcopenia, whereas those whose PEFR improved from <80% to ≥80% showed a 30% reduction in risk. This asymmetrical association may reflect ongoing muscular degradation occurring alongside declining PEFR—potentially driven by shared pathophysiological mechanisms such as systemic inflammation, reduced physical activity, or impaired energy metabolism. Even if PEFR later recovers, the regenerative capacity of damaged muscle fibers is often limited, and mitochondrial function may not recover in parallel, making it difficult to fully reverse muscle mass and function. These findings underscore the potential value of monitoring PEFR trajectories in older adults as part of routine assessment, where early detection of decline may provide a more favorable opportunity for sarcopenia prevention than interventions initiated after marked functional deterioration.

This study has several strengths. First, it is the first to investigate the association between longitudinal changes in PEFR and the subsequent risk of sarcopenia, thereby extending current understanding beyond baseline measurements. Second, the use of a nationally representative cohort of community-dwelling older adults enhances the external validity and generalizability of the findings. Finally, by integrating both cross-sectional and longitudinal analyses, the study offers a comprehensive understanding of how baseline and changing levels of PEFR relate to sarcopenia risk, with adjustment for multiple confounders, strengthening the robustness of the observed association.

The limitations of this study must be acknowledged. First, ASM mass was estimated using an anthropometric equation, which may not align exactly with DXA measurements. Nevertheless, research by Alexandre et al. [40] has demonstrated that, when combined with handgrip strength and gait speed, anthropometric equations can serve as an effective and cost-effective alternative to DXA for diagnosing sarcopenia. The formula we employed, developed by Wen et al. [41] for the Chinese population, showed an adjusted R² of 0.90 and a standard error of 1.63 kg in comparison to DXA. Although simpler than DXA, this method is practical, especially in resource-limited settings. Indeed, it has been validated in nearly 100 studies, further confirming its reliability. Additionally, due to the tendency for random errors to cancel each other out in large-scale studies, the bias—both in direction and magnitude—has minimal impact on the overall results. Second, despite adjusting for multiple potential confounders in the multivariable analysis, some factors, such as nutritional status and physical activity level, both of which are pivotal in the development of sarcopenia, could not be fully accounted for due to missing data. To reflect nutritional status, we used BMI, digestive diseases, and complete tooth loss as proxies; for physical activity level, we included the 6-item ADL scale and physical activities like going to parks to practice Tai Chi to approximate its effect. Although these limitations exist, this study adds to our understanding of the significance of changes in PEFR status in relation to sarcopenia.

## 5 Conclusions

In conclusion, this study demonstrates that both lower baseline PEFR and declines over time are independently associated with increased risk of sarcopenia in older adults, while improvements in PEFR are linked to reduced risk. These findings suggest that PEFR, a simple and non-invasive pulmonary function measure, may serve as a practical marker for early identification of individuals at elevated risk. Incorporating PEFR monitoring into routine geriatric assessments could support timely preventive strategies for sarcopenia, particularly in resource-limited settings.

## Clinical trial number

Not applicable.

## CRediT authorship contribution statement

Study design: CKK. Data analysis: QH and CQF. Result interpretation: QH. Reporting & editing: QH and CKK. Final approval of the version to be submitted: CKK, QH, and CQF.

## Consent to participate

All participants signed an informed consent.

## Consent for publication

Not applicable.

## Ethical approval

CHARLS study is an open dataset. The CHARLS was approved by the Ethics Review Committee of Peking University (IRB 00001052-11015).

## Statement of human and animal rights

CHARLS study is an open dataset. All procedures performed in CHARLS study involving human participants were in accordance with the ethical standards of the Ethics Review Committee of Peking University (IRB 00001052–11015) and with the 1964 Helsinki declaration and its later amendments or comparable ethical standards.

## Funding

The study has not been sponsored.

## Data availability

Details of how to access the CHARLS data and details of the data release schedule are available from https://charls.charlsdata.com/index/zh-cn.html.

## Declaration of competing interest

The authors declare that they have no conflict of interests.
