# Supplementary material for: Baseline and longitudinal changes in peak expiratory flow rate as predictors of sarcopenia in older adults: A 4-year cohort study
Source: J Nutr Health Aging. 2025 Jul 24;29(9):100640. doi: 10.1016/j.jnha.2025.100640 (PMC12311495; doi:10.1016/j.jnha.2025.100640)
Supplement: Supplementary file 1 [file mmc1.docx]

Table S1. Association between PEFR (per 1 SD decrease) and prevalent sarcopenia across sex- and age-defined subgroups (n=5,280).

| **Subgroup** | Prevalent sarcopenia | | | | |
| --- | --- | --- | --- | --- | --- |
|  | Model 1 | |  | Model 2 | |
|  | OR (95% CI) | *p*-value |  | OR (95% CI) | *p*-value |
|  |  |  |  |  |  |
| **Male <75 years** (n=2,285) |  |  |  |  |  |
| PEFR continuous (1 SD decrease) | 1.46 (1.25-1.70) | **<0.001** |  | 1.42 (1.16-1.74) | **<0.001** |
| **Male ≥75 years** (n=429) |  |  |  |  |  |
| PEFR continuous (1 SD decrease) | 1.46 (1.17-1.84) | **<0.001** |  | 1.43 (1.01-2.02) | **0.044** |
| **Female <75 years** (n=2,137) |  |  |  |  |  |
| PEFR continuous (1 SD decrease) | 1.56 (1.37-1.79) | **<0.001** |  | 1.51 (1.29-1.78) | **<0.001** |
| **Female ≥75 years** (n=429) |  |  |  |  |  |
| PEFR continuous (1 SD decrease) | 1.71 (1.36-2.14) | **<0.001** |  | 1.84 (1.37-2.47) | **<0.001** |

PEFR, peak expiratory flow rate; OR, odds ratio; CI, confidence interval; SD, standard deviation

Model 1 adjusted for age, marital status, education levels, type of residence, smoking, alcohol consumption, physical activities, complete tooth loss, and activities of daily living;

Model 2 adjusted for model 1 plus body mass index, chronic lung disease, asthma, diabetes, heart problem, stroke, cancer, kidney disease, liver disease, arthritis, hypertension, digestive disease, and number of medications.
