# Supplementary material for: Baseline and longitudinal changes in peak expiratory flow rate as predictors of sarcopenia in older adults: A 4-year cohort study
Source: J Nutr Health Aging. 2025 Jul 24;29(9):100640. doi: 10.1016/j.jnha.2025.100640 (PMC12311495; doi:10.1016/j.jnha.2025.100640)
Supplement: Supplementary file 2 [file mmc2.docx]

Table S2. Cross-sectional analysis of the influences of the interactions between peak expiratory flow rate (%predicted) and potential confounders on sarcopenia (N=5,280).

| Variables | n (%) | PEFR (%predicted) ≥80%  sarcopenia/total | PEFR (%predicted) <80%  sarcopenia/total | OR (95%CI) | *P* | *P* for interaction |
| --- | --- | --- | --- | --- | --- | --- |
| All patients (%) | 5,280 (100.00) | 137/1,157 | 871/4,123 | 1.93 (1.49 ~ 2.50) | <.001 |  |
| Age group (%) |  |  |  |  |  | 0.105 |
| 60-69 | 3,498 (66.25) | 44/789 | 355/2,709 | 2.22 (1.59 ~ 3.10) | **<.001** |  |
| 70-79 | 1,498 (28.37) | 68/304 | 380/1,194 | 1.42 (1.04 ~ 1.94) | **0.029** |  |
| ≥ 80 | 284 (5.38) | 25/64 | 136/220 | 2.94 (1.49 ~ 5.77) | **0.002** |  |
| Sex (%) |  |  |  |  |  | 0.877 |
| Men | 2,714 (51.40) | 48/575 | 375/2,139 | 1.85 (1.31 ~ 2.60) | **<.001** |  |
| Women | 2,566 (48.60) | 89/582 | 496/1,984 | 1.90 (1.44 ~ 2.50) | **<.001** |  |
| Residential area (%) |  |  |  |  |  | 0.342 |
| Urban area | 1,944 (36.82) | 46/479 | 231/1,465 | 1.63 (1.11 ~ 2.40) | **0.012** |  |
| Rural area | 3,336 (63.18) | 91/678 | 640/2,658 | 2.00 (1.54 ~ 2.59) | **<.001** |  |
| Marital status (%) |  |  |  |  |  | 0.362 |
| Married/married but separated | 4,206 (79.66) | 84/935 | 591/3,271 | 2.00 (1.55 ~ 2.59) | **<.001** |  |
| Unmarried/divorced/widowed | 1,074 (20.34) | 53/222 | 280/852 | 1.65 (1.13 ~ 2.43) | **0.010** |  |
| Education level (%) |  |  |  |  |  | 0.218 |
| No formal education | 2,971 (56.27) | 97/541 | 647/2,430 | 1.90 (1.46 ~ 2.47) | **<.001** |  |
| Primary school | 1,369 (25.93) | 32/324 | 164/1,045 | 1.57 (1.03 ~ 2.40) | **0.037** |  |
| Middle school | 621 (11.76) | 7/174 | 42/447 | 2.03 (0.84 ~ 4.91) | 0.118 |  |
| High school or above | 319 (6.04) | 1/118 | 18/201 | 12.02 (1.12 ~ 128.56) | **0.040** |  |
| Smoking (%) |  |  |  |  |  | 0.536 |
| No | 2,997 (56.76) | 95/704 | 510/2,293 | 1.80 (1.38 ~ 2.35) | **<.001** |  |
| Yes | 2,283 (43.24) | 42/453 | 361/1,830 | 2.00 (1.39 ~ 2.89) | **<.001** |  |
| Alcohol consumption (%) |  |  |  |  |  | 0.995 |
| No | 3,124 (59.17) | 96/709 | 556/2,415 | 1.86 (1.43 ~ 2.42) | **<.001** |  |
| Yes | 2,156 (40.83) | 41/448 | 315/1,708 | 1.88 (1.29 ~ 2.72) | **<.001** |  |
| Chronic lung disease (%) |  |  |  |  |  | 0.262 |
| No | 4,575 (86.65) | 131/1,093 | 712/3,482 | 1.80 (1.44 ~ 2.24) | **<.001** |  |
| Yes | 705 (13.35) | 6/64 | 159/641 | 3.12 (1.21 ~ 8.05) | **0.019** |  |
| Asthma (%) |  |  |  |  |  | 0.174 |
| No | 4,938 (93.52) | 136/1,140 | 792/3,798 | 1.83 (1.48 ~ 2.27) | **<.001** |  |
| Yes | 342 (6.48) | 1/17 | 79/325 | 8.02 (0.81 ~ 79.50) | 0.075 |  |
| Diabetes (%) |  |  |  |  |  | 0.338 |
| No | 4,877 (92.37) | 134/1,060 | 838/3,817 | 1.82 (1.47 ~ 2.26) | **<.001** |  |
| Yes | 403 (7.63) | 3/97 | 33/306 | 13.61 (2.54 ~ 72.78) | **0.002** |  |
| Heart Problem (%) |  |  |  |  |  | 0.242 |
| No | 4,444 (84.17) | 119/989 | 767/3,455 | 1.95 (1.55 ~ 2.45) | **<.001** |  |
| Yes | 836 (15.83) | 18/168 | 104/668 | 1.43 (0.78 ~ 2.61) | 0.242 |  |
| Stroke (%) |  |  |  |  |  | 0.502 |
| No | 5,090 (96.40) | 130/1,124 | 838/3,966 | 1.89 (1.52 ~ 2.35) | **<.001** |  |
| Yes | 190 (3.60) | 7/33 | 33/157 | 1.75 (0.39 ~ 7.80) | 0.460 |  |
| Kidney diseases (%) |  |  |  |  |  | 0.600 |
| No | 4,971 (94.15) | 131/1,102 | 826/3,869 | 1.88 (1.51 ~ 2.34) | **<.001** |  |
| Yes | 309 (5.85) | 6/55 | 45/254 | 1.74 (0.62 ~ 4.90) | 0.296 |  |
| Liver diseases (%) |  |  |  |  |  | 0.837 |
| No | 5,102 (96.63) | 134/1,124 | 848/3,978 | 1.88 (1.52 ~ 2.33) | **<.001** |  |
| Yes | 178 (3.37) | 3/33 | 23/145 | 1.56 (0.29 ~ 8.47) | 0.608 |  |
| Arthritis (%) |  |  |  |  |  | 0.377 |
| No | 3,324 (62.95) | 81/721 | 566/2,603 | 2.00 (1.52 ~ 2.64) | **<.001** |  |
| Yes | 1,956 (37.05) | 56/436 | 305/1,520 | 1.71 (1.22 ~ 2.39) | **0.002** |  |
| Hypertension (%) |  |  |  |  |  | 0.815 |
| No | 3,538 (67.01) | 104/764 | 669/2,774 | 1.90 (1.49 ~ 2.44) | **<.001** |  |
| Yes | 1,742 (32.99) | 33/393 | 202/1,349 | 1.70 (1.12 ~ 2.60) | **0.013** |  |
| Digestive disease (%) |  |  |  |  |  | 0.770 |
| No | 4,139 (78.39) | 107/926 | 662/3,213 | 1.87 (1.47 ~ 2.38) | **<.001** |  |
| Yes | 1,141 (21.61) | 30/231 | 209/910 | 1.84 (1.16 ~ 2.90) | **0.009** |  |
| Number of medications (%) |  |  |  |  |  | 0.437 |
| 0 | 2,409 (45.62) | 69/563 | 431/1,846 | 2.08 (1.52 ~ 2.83) | **<.001** |  |
| 1 | 1,592 (30.15) | 47/367 | 244/1,225 | 1.50 (1.04 ~ 2.17) | **0.031** |  |
| ≥2 | 1,279 (24.22) | 21/227 | 196/1,052 | 2.07 (1.22 ~ 3.49) | **0.007** |  |
| Activities of daily living (%) |  |  |  |  |  | 0.367 |
| 0 | 4,082 (77.31) | 102/966 | 586/3,116 | 1.86 (1.45 ~ 2.38) | **<.001** |  |
| 1 | 626 (11.86) | 20/107 | 130/519 | 1.53 (0.86 ~ 2.73) | 0.149 |  |
| ≥2 | 572 (10.83) | 15/84 | 155/488 | 2.63 (1.36 ~ 5.12) | **0.004** |  |
| Complete tooth loss (%) |  |  |  |  |  | 0.299 |
| No | 4,419 (83.69) | 103/1,008 | 614/3,411 | 1.77 (1.39 ~ 2.24) | **<.001** |  |
| Yes | 861 (16.31) | 34/149 | 257/712 | 2.23 (1.40 ~ 3.55) | **<.001** |  |
| Physical activities (%) |  |  |  |  |  | 0.411 |
| No | 2,510 (47.54) | 64/607 | 354/1,903 | 1.73 (1.26 ~ 2.37) | **<.001** |  |
| Yes | 2,770 (52.46) | 73/550 | 517/2,220 | 2.02 (1.51 ~ 2.71) | **<.001** |  |

PEFR, peak expiratory flow rate; *OR, odds ratio; CI, confidence intervals.*
