# Supplementary material for: Baseline and longitudinal changes in peak expiratory flow rate as predictors of sarcopenia in older adults: A 4-year cohort study
Source: J Nutr Health Aging. 2025 Jul 24;29(9):100640. doi: 10.1016/j.jnha.2025.100640 (PMC12311495; doi:10.1016/j.jnha.2025.100640)
Supplement: Supplementary file 3 [file mmc3.docx]

Table S3. Association between PEFR (per 1 SD Decrease) and incident sarcopenia across sex- and age-defined subgroups (n=3,686)

| **Subgroup** |  | Incident sarcopenia | | | | |
| --- | --- | --- | --- | --- | --- | --- |
|  |  | Model 1 | |  | Model 2 | |
|  |  | HR (95% CI) | *p*-value |  | HR (95% CI) | *p*-value |
|  |  |  |  |  |  |  |
| **Male <75 years** (n=1,771) |  |  |  |  |  |  |
| PEFR continuous (1 SD decrease) |  | 1.28 (1.10-1.50) | **0.002** |  | 1.21 (1.02-1.44) | **0.027** |
| **Male ≥75 years** (n=190) |  |  |  |  |  |  |
| PEFR continuous (1 SD decrease) |  | 1.11 (0.80-1.53) | 0.543 |  | 1.16 (0.79-1.71) | 0.451 |
| **Female <75 years** (n=1,557) |  |  |  |  |  |  |
| PEFR continuous (1 SD decrease) |  | 1.34 (1.16-1.54) | **<0.001** |  | 1.27 (1.10-1.46) | **0.001** |
| **Female ≥75 years** (n=168) |  |  |  |  |  |  |
| PEFR continuous (1 SD decrease) |  | 1.15 (0.87-1.51) | 0.325 |  | 1.23 (0.92-1.66) | 0.167 |

PEFR, peak expiratory flow rate; HR, hazard ratio; CI, confidence interval; SD, standard deviation

Model 1 adjusted for age, marital status, education levels, type of residence, smoking, alcohol consumption, physical activities, complete tooth loss, and activities of daily living;

Model 2 adjusted for model 1 plus body mass index, chronic lung disease, asthma, diabetes, heart problem, stroke, cancer, kidney disease, liver disease, arthritis, hypertension, digestive disease, and number of medications.
