# Supplementary material for: Baseline and longitudinal changes in peak expiratory flow rate as predictors of sarcopenia in older adults: A 4-year cohort study
Source: J Nutr Health Aging. 2025 Jul 24;29(9):100640. doi: 10.1016/j.jnha.2025.100640 (PMC12311495; doi:10.1016/j.jnha.2025.100640)
Supplement: Supplementary file 4 [file mmc4.docx]

Table S4. Longitudinal analysis of the influences of the interactions between peak expiratory flow rate (%predicted) and potential confounders on sarcopenia (N=3,686).

| Variables | n (%) | PEFR (%predicted) ≥80%  sarcopenia/total | PEFR (%predicted) <80%  sarcopenia/total | HR (95%CI) | *P* | *P* for interaction |
| --- | --- | --- | --- | --- | --- | --- |
| All patients (%) | 3,686 (100.00) | 92/898 | 433/2,788 | 1.47 (1.17 ~ 1.84) | <.001 |  |
| Age group (%) |  |  |  |  |  | 0.276 |
| 60-69 | 2,735 (74.20) | 43/668 | 242/2,067 | 1.64 (1.18 ~ 2.29) | **0.003** |  |
| 70-79 | 872 (23.66) | 41/203 | 172/669 | 1.22 (0.86 ~ 1.73) | 0.266 |  |
| ≥ 80 | 79 (2.14) | 8/27 | 19/52 | 1.27 (0.43 ~ 3.73) | 0.665 |  |
| Sex (%) |  |  |  |  |  | 0.806 |
| Men | 1,961 (53.20) | 37/464 | 196/1,497 | 1.46 (1.02 ~ 2.10) | **0.038** |  |
| Women | 1,725 (46.80) | 55/434 | 237/1,291 | 1.36 (1.01 ~ 1.84) | **0.042** |  |
| Residential area (%) |  |  |  |  |  | **0.005** |
| Urban area | 1,345 (36.49) | 14/359 | 112/986 | 2.70 (1.53 ~ 4.77) | **<.001** |  |
| Rural area | 2,341 (63.51) | 78/539 | 321/1,802 | 1.18 (0.91 ~ 1.51) | 0.208 |  |
| Marital status (%) |  |  |  |  |  | 0.054 |
| Married/married but separated | 3,069 (83.26) | 66/756 | 341/2,313 | 1.54 (1.17 ~ 2.01) | **0.002** |  |
| Unmarried/divorced/widowed | 617 (16.74) | 26/142 | 92/475 | 0.93 (0.59 ~ 1.46) | 0.752 |  |
| Education level (%) |  |  |  |  |  | 0.191 |
| No formal education | 1,933 (52.44) | 64/397 | 291/1,536 | 1.17 (0.89 ~ 1.55) | 0.254 |  |
| Primary school | 1,038 (28.16) | 19/271 | 97/767 | 2.00 (1.21 ~ 3.31) | **0.007** |  |
| Middle school | 487 (13.21) | 6/143 | 26/344 | 1.67 (0.64 ~ 4.36) | 0.293 |  |
| High school or above | 228 (6.19) | 3/87 | 19/141 | 3.14 (0.85 ~ 11.67) | 0.087 |  |
| Smoking (%) |  |  |  |  |  | 0.270 |
| No | 2,088 (56.65) | 64/543 | 249/1,545 | 1.27 (0.96 ~ 1.68) | 0.098 |  |
| Yes | 1,598 (43.35) | 28/355 | 184/1,243 | 1.77 (1.18 ~ 2.67) | **0.006** |  |
| Alcohol consumption (%) |  |  |  |  |  | 0.680 |
| No | 2,143 (58.14) | 59/539 | 272/1,604 | 1.44 (1.08 ~ 1.91) | **0.013** |  |
| Yes | 1,543 (41.86) | 33/359 | 161/1,184 | 1.31 (0.89 ~ 1.93) | 0.170 |  |
| Chronic lung disease (%) |  |  |  |  |  | 0.490 |
| No | 3,226 (87.52) | 88/850 | 353/2,376 | 1.38 (1.09 ~ 1.75) | **0.007** |  |
| Yes | 460 (12.48) | 4/48 | 80/412 | 1.85 (0.65 ~ 5.22) | 0.246 |  |
| Asthma (%) |  |  |  |  |  | 0.499 |
| No | 3,463 (93.95) | 91/886 | 388/2,577 | 1.39 (1.10 ~ 1.75) | **0.005** |  |
| Yes | 223 (6.05) | 1/12 | 45/211 | 1.36 (0.16 ~ 11.33) | 0.776 |  |
| Diabetes (%) |  |  |  |  |  | 0.155 |
| No | 3,389 (91.94) | 90/818 | 411/2,571 | 1.36 (1.08 ~ 1.72) | **0.010** |  |
| Yes | 297 (8.06) | 2/80 | 22/217 | 2.61 (0.58 ~ 11.89) | 0.214 |  |
| Heart Problem (%) |  |  |  |  |  | 0.739 |
| No | 3,116 (84.54) | 82/774 | 364/2,342 | 1.41 (1.11 ~ 1.80) | **0.005** |  |
| Yes | 570 (15.46) | 10/124 | 69/446 | 1.28 (0.63 ~ 2.61) | 0.490 |  |
| Stroke (%) |  |  |  |  |  | 0.368 |
| No | 3,568 (96.80) | 91/876 | 422/2,692 | 1.38 (1.09 ~ 1.74) | **0.006** |  |
| Yes | 118 (3.20) | 1/22 | 11/96 | 5.83 (0.24 ~ 140.97) | 0.278 |  |
| Kidney diseases (%) |  |  |  |  |  | 0.377 |
| No | 3,457 (93.79) | 90/855 | 406/2,602 | 1.37 (1.08 ~ 1.73) | **0.008** |  |
| Yes | 229 (6.21) | 2/43 | 27/186 | 3.01 (0.68 ~ 13.38) | 0.149 |  |
| Arthritis (%) |  |  |  |  |  | 0.613 |
| No | 2,304 (62.51) | 54/565 | 269/1,739 | 1.50 (1.11 ~ 2.01) | **0.008** |  |
| Yes | 1,382 (37.49) | 38/333 | 164/1,049 | 1.19 (0.83 ~ 1.72) | 0.348 |  |
| Hypertension (%) |  |  |  |  |  | 0.825 |
| No | 2,433 (66.01) | 67/589 | 321/1,844 | 1.43 (1.09 ~ 1.87) | **0.009** |  |
| Yes | 1,253 (33.99) | 25/309 | 112/944 | 1.38 (0.88 ~ 2.16) | 0.155 |  |
| Digestive disease (%) |  |  |  |  |  | 0.087 |
| No | 2,896 (78.57) | 67/718 | 335/2,178 | 1.57 (1.21 ~ 2.06) | **<.001** |  |
| Yes | 790 (21.43) | 25/180 | 98/610 | 1.03 (0.65 ~ 1.64) | 0.897 |  |
| Number of medications (%) |  |  |  |  |  | **0.010** |
| 0 | 1,703 (46.20) | 35/449 | 215/1,254 | 2.00 (1.40 ~ 2.88) | **<.001** |  |
| 1 | 1,119 (30.36) | 38/279 | 119/840 | 0.98 (0.67 ~ 1.43) | 0.910 |  |
| ≥2 | 864 (23.44) | 19/170 | 99/694 | 1.10 (0.65 ~ 1.86) | 0.712 |  |
| Activities of daily living (%) |  |  |  |  |  | 0.566 |
| 0 | 2,954 (80.14) | 74/764 | 335/2,190 | 1.46 (1.13 ~ 1.88) | **0.004** |  |
| 1 | 405 (10.99) | 8/76 | 48/329 | 1.51 (0.70 ~ 3.27) | 0.290 |  |
| ≥2 | 327 (8.87) | 10/58 | 50/269 | 0.86 (0.41 ~ 1.80) | 0.693 |  |
| Complete tooth loss (%) |  |  |  |  |  | 0.194 |
| No | 3,194 (86.65) | 71/797 | 348/2,397 | 1.49 (1.15 ~ 1.93) | **0.003** |  |
| Yes | 492 (13.35) | 21/101 | 85/391 | 1.20 (0.72 ~ 2.00) | 0.477 |  |
| Physical activities (%) |  |  |  |  |  | 0.600 |
| No | 1,811 (49.13) | 47/477 | 192/1,334 | 1.33 (0.96 ~ 1.85) | 0.084 |  |
| Yes | 1,875 (50.87) | 45/421 | 241/1,454 | 1.47 (1.06 ~ 2.04) | **0.020** |  |

PEFR, peak expiratory flow rate; *HR, hazard ratio; CI, confidence intervals.*
