# Supplementary material for: Baseline and longitudinal changes in peak expiratory flow rate as predictors of sarcopenia in older adults: A 4-year cohort study
Source: J Nutr Health Aging. 2025 Jul 24;29(9):100640. doi: 10.1016/j.jnha.2025.100640 (PMC12311495; doi:10.1016/j.jnha.2025.100640)
Supplement: Supplementary file 5 [file mmc5.docx]

Table S5. The influences of the interactions between changes in peak expiratory flow rate (%predicted) and potential confounders on sarcopenia (N=2,189).

| Variables | n (%) | Stable PEFR (%predicted) ≥80%  sarcopenia/total | PEFR (%predicted) <80% to ≥80%  sarcopenia/total | HR (95%CI) | *P* | *P* for interaction |
| --- | --- | --- | --- | --- | --- | --- |
| All patients (%) | 2,189 (100.00) | 299/1,761 | 44/428 | 0.70 (0.50-0.96) | **0.028** |  |
| Age group (%) |  |  |  |  |  | 0.352 |
| 60-69 | 1,618 (73.92) | 168/1,292 | 23/326 | 0.46 (0.23 ~ 0.91) | **0.026** |  |
| 70-79 | 532 (24.30) | 119/433 | 20/99 | 0.72 (0.33 ~ 1.55) | 0.395 |  |
| ≥ 80 | 39 (1.78) | 12/36 | 1/3 | 0.00 (0.00 ~ Inf) | 1.000 |  |
| Sex (%) |  |  |  |  |  | 0.799 |
| Men | 1,196 (54.64) | 144/985 | 14/211 | 0.53 (0.26 ~ 1.07) | 0.075 |  |
| Women | 993 (45.36) | 155/776 | 30/217 | 0.53 (0.26 ~ 1.10) | 0.088 |  |
| Residential area (%) |  |  |  |  |  | 0.680 |
| Urban area | 745 (34.03) | 74/559 | 15/186 | 0.54 (0.31 ~ 0.96) | **0.037** |  |
| Rural area | 1,444 (65.97) | 225/1,202 | 29/242 | 0.64 (0.43 ~ 0.94) | **0.025** |  |
| Marital status (%) |  |  |  |  |  | 0.546 |
| Married/married but separated | 1,823 (83.28) | 236/1,464 | 34/359 | 0.60 (0.41 ~ 0.86) | **0.006** |  |
| Unmarried/divorced/widowed | 366 (16.72) | 63/297 | 10/69 | 0.79 (0.38 ~ 1.64) | 0.531 |  |
| Education level (%) |  |  |  |  |  | 0.540 |
| No formal education | 1,194 (54.55) | 201/981 | 29/213 | 0.66 (0.45 ~ 0.99) | **0.042** |  |
| Primary school | 615 (28.10) | 69/493 | 11/122 | 0.56 (0.29 ~ 1.08) | 0.081 |  |
| Middle school | 272 (12.43) | 20/210 | 1/62 | 0.10 (0.01 ~ 1.06) | 0.056 |  |
| High school or above | 108 (4.93) | 9/77 | 3/31 | 0.46 (0.04 ~ 5.28) | 0.534 |  |
| Smoking (%) |  |  |  |  |  | 0.692 |
| No | 1,203 (54.96) | 172/942 | 28/261 | 0.62 (0.41 ~ 0.93) | **0.021** |  |
| Yes | 986 (45.04) | 127/819 | 16/167 | 0.61 (0.36 ~ 1.05) | 0.074 |  |
| Alcohol consumption (%) |  |  |  |  |  | 0.767 |
| No | 1,259 (57.51) | 184/987 | 32/272 | 0.63 (0.43 ~ 0.93) | **0.020** |  |
| Yes | 930 (42.49) | 115/774 | 12/156 | 0.55 (0.30 ~ 1.01) | 0.054 |  |
| Chronic lung disease (%) |  |  |  |  |  | 0.504 |
| No | 1,875 (85.66) | 247/1,479 | 40/396 | 0.60 (0.43 ~ 0.85) | **0.004** |  |
| Yes | 314 (14.34) | 52/282 | 4/32 | 0.99 (0.33 ~ 2.99) | 0.988 |  |
| Asthma (%) |  |  |  |  |  | 0.991 |
| No | 2,028 (92.65) | 266/1,606 | 44/422 | 0.63 (0.46 ~ 0.87) | **0.005** |  |
| Yes | 161 (7.35) | 33/155 | 0/6 | 0.00 (0.00 ~ Inf) | 0.997 |  |
| Diabetes (%) |  |  |  |  |  | 0.444 |
| No | 2,010 (91.82) | 281/1,626 | 42/384 | 0.64 (0.46 ~ 0.89) | **0.008** |  |
| Yes | 179 (8.18) | 18/135 | 2/44 | 0.31 (0.05 ~ 1.90) | 0.206 |  |
| Heart Problem (%) |  |  |  |  |  | 0.779 |
| No | 1,849 (84.47) | 250/1,488 | 38/361 | 0.62 (0.44 ~ 0.88) | **0.008** |  |
| Yes | 340 (15.53) | 49/273 | 6/67 | 0.57 (0.22 ~ 1.46) | 0.241 |  |
| Kidney diseases (%) |  |  |  |  |  | 0.197 |
| No | 2,042 (93.28) | 280/1,633 | 41/409 | 0.60 (0.43 ~ 0.84) | **0.003** |  |
| Yes | 147 (6.72) | 19/128 | 3/19 | 0.83 (0.17 ~ 4.07) | 0.820 |  |
| Arthritis (%) |  |  |  |  |  | 0.585 |
| No | 1,353 (61.81) | 183/1,094 | 29/259 | 0.64 (0.43 ~ 0.96) | **0.032** |  |
| Yes | 836 (38.19) | 116/667 | 15/169 | 0.57 (0.33 ~ 0.99) | 0.047 |  |
| Hypertension (%) |  |  |  |  |  | 0.989 |
| No | 1,458 (66.61) | 221/1,163 | 34/295 | 0.62 (0.43 ~ 0.90) | **0.011** |  |
| Yes | 731 (33.39) | 78/598 | 10/133 | 0.59 (0.30 ~ 1.17) | 0.129 |  |
| Digestive disease (%) |  |  |  |  |  | 0.161 |
| No | 1,696 (77.48) | 236/1,366 | 29/330 | 0.55 (0.37 ~ 0.81) | **0.003** |  |
| Yes | 493 (22.52) | 63/395 | 15/98 | 0.80 (0.43 ~ 1.47) | 0.472 |  |
| Number of medications (%) |  |  |  |  |  | 0.301 |
| 0 | 985 (45.00) | 155/793 | 20/192 | 0.55 (0.34 ~ 0.88) | **0.013** |  |
| 1 | 659 (30.11) | 76/512 | 17/147 | 0.78 (0.45 ~ 1.35) | 0.378 |  |
| ≥2 | 545 (24.90) | 68/456 | 7/89 | 0.56 (0.25 ~ 1.27) | 0.164 |  |
| Activities of daily living (%) |  |  |  |  |  | 0.383 |
| 0 | 1,724 (78.76) | 227/1,371 | 39/353 | 0.70 (0.49 ~ 0.98) | **0.041** |  |
| 1 | 262 (11.97) | 35/216 | 3/46 | 0.33 (0.09 ~ 1.16) | 0.084 |  |
| ≥2 | 203 (9.27) | 37/174 | 2/29 | 0.26 (0.06 ~ 1.15) | 0.076 |  |
| Complete tooth loss (%) |  |  |  |  |  | 0.532 |
| No | 1,880 (85.88) | 235/1,502 | 37/378 | 0.65 (0.46 ~ 0.93) | **0.019** |  |
| Yes | 309 (14.12) | 64/259 | 7/50 | 0.52 (0.23 ~ 1.17) | 0.114 |  |
| Physical activities (%) |  |  |  |  |  | 0.659 |
| No | 1,065 (48.65) | 140/854 | 19/211 | 0.57 (0.35 ~ 0.94) | **0.027** |  |
| Yes | 1,124 (51.35) | 159/907 | 25/217 | 0.63 (0.41 ~ 0.98) | **0.038** |  |

*PEFR, peak expiratory flow rate; HR, hazard ratio; CI, confidence intervals.*
