# Supplementary figures and images for: Baseline and longitudinal changes in peak expiratory flow rate as predictors of sarcopenia in older adults: A 4-year cohort study
Source: J Nutr Health Aging. 2025 Jul 24;29(9):100640. doi: 10.1016/j.jnha.2025.100640 (PMC12311495; doi:10.1016/j.jnha.2025.100640)

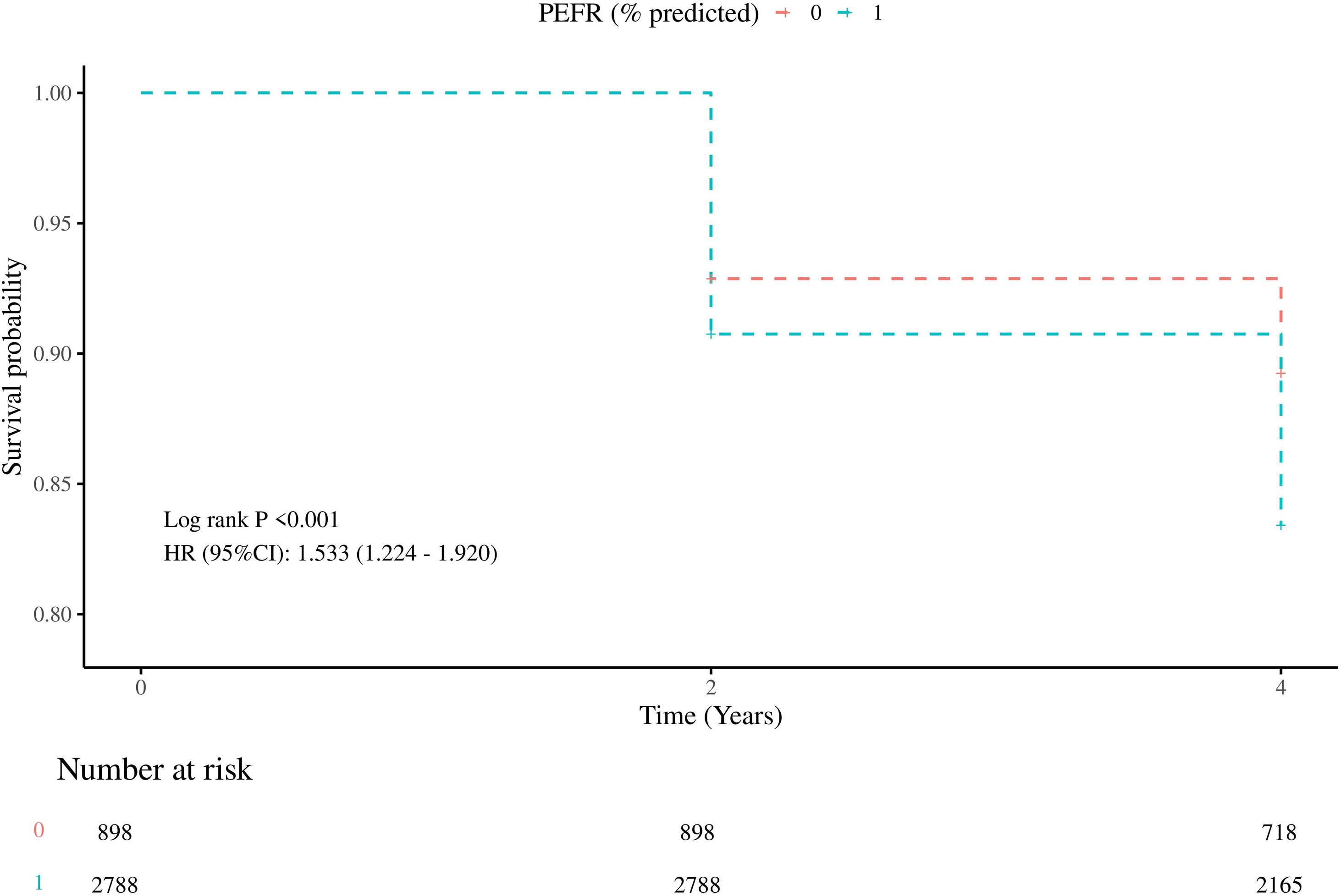

Supplement: Supplementary file 6 [file mmc6.jpg]
